# Harnessing B7-H6 for Anticancer Immunotherapy: Expression, Pathways, and Therapeutic Strategies

**DOI:** 10.3390/ijms251910326

**Published:** 2024-09-25

**Authors:** Sunyoung Lee, Ji Hyun Kim, In-Hwan Jang, Seona Jo, Soo Yun Lee, Se-Chan Oh, Seok-Min Kim, Lingzu Kong, Jesang Ko, Tae-Don Kim

**Affiliations:** 1 Center for Cell and Gene Therapy, Korea Research Institute of Bioscience and Biotechnology (KRIBB), Daejeon 34141, Republic of Korea; christy0925@kribb.re.kr (S.L.); jihyun3554@kribb.re.kr (J.H.K.); jangih@kribb.re.kr (I.-H.J.); seona423@kribb.re.kr (S.J.); sooyun27@kribb.re.kr (S.Y.L.); medic305@kribb.re.kr (S.-C.O.); okwwwitv@kribb.re.kr (S.-M.K.); konglingzu93@kribb.re.kr (L.K.); 2 Division of Life Sciences, Korea University, Seoul 02841, Republic of Korea; jesangko@korea.ac.kr; 3 KRIBB School of Bioscience, Korea University of Science and Technology (UST), Daejeon 34113, Republic of Korea; 4 Department of Biochemistry, College of Natural Sciences, Chungnam National University, Daejeon 34134, Republic of Korea

**Keywords:** B7-H6, NKp30, cancer-specific target, targeted therapy

## Abstract

Cancer therapies have evolved from traditional chemotherapy to more precise molecular-targeted immunotherapies, which have been associated with improved side effects and outcomes. These modern strategies rely on cancer-specific biomarkers that differentiate malignant from normal cells. The B7 family of immune checkpoint molecules is crucial for cancer immune evasion and a prime therapeutic target. B7-H6, a recently identified member of the B7 family, has emerged as a promising therapeutic target. Unlike other B7 proteins, B7-H6 is not expressed in healthy tissues but is upregulated in several cancers. It binds to NKp30, activating natural killer (NK) cells and triggering immune responses against cancer cells. This review explores the expression of B7-H6 in different cancers, the factors that regulate its expression, and its intrinsic and extrinsic pathways. Additionally, we discuss potential anticancer therapies targeting B7-H6, highlighting its significance in advancing precision medicine. Understanding the role of B7-H6 in cancer immunity may inform the development of appropriate therapies that exploit its cancer-specific expression.

## 1. Introduction

Cancer is one of the most important human diseases to overcome. Recently, cancer treatments have progressed from merely improving survival rates to reducing side effects and preventing recurrence. Early cancer treatments relied heavily on chemical anticancer drugs that were limited by their adverse effects on normal tissues, which has led to the development of cancer-specific targeted therapies. Molecular targeted therapies, including small molecules and therapeutic monoclonal antibodies, were first approved for clinical use in the late 1990s and have since become the foundation of precision medicine [1]. Compared with conventional chemotherapy, these therapies offer fewer side effects and improved efficacy. Immunotherapy, which enhances the body’s natural defenses to eliminate cancer cells, has also made great advances [2]. These include oncolytic viral therapies, cancer vaccines, cytokine therapies, adoptive cell transfer, and immune checkpoint inhibitors. Immunotherapies also provide greater safety and fewer side effects by utilizing the host immune system.

The precision of both molecular targeted and immune therapies relies on the identification of cancer-specific biomarkers. The B7 family of immune checkpoint molecules plays a crucial role in cancer immune evasion [3,4], making them prime therapeutic targets. PD-1 and its ligand PD-L1 are the best-known immune checkpoint inhibitors, with drugs such as nivolumab, pembrolizumab, and atezolizumab approved for clinical use in various cancers [5,6].

B7-H6, one of the members of the B7 family, is a costimulatory or co-inhibitory molecule that was identified in 2009 by Brandt et al. [7,8]. Unlike other B7 members, B7-H6 is rarely expressed in normal tissues but is upregulated in various cancers. It interacts with NKp30, an activating receptor on natural killer (NK) cells, triggering innate immune responses. B7-H6 is a 51 kD type-1 transmembrane protein composed of 454 amino acids [7]. Similar to other B7 family members, B7-H6 has two immunoglobulin (Ig) domains, an IgC domain, an IgV domain, and a signal peptide. Unlike other B7 family members, it has a homologous glycosaminoglycan (GAG) polyprotein in its intracytoplasmic domain. Interestingly, B7-H6 has an immunoreceptor tyrosine-based inhibition motif-like domain (SaYtpL) and Src homology 2 (SH2) and Src homology 3 (SH3)-binding motifs, which facilitate intracellular signaling. The gene sequence of B7-H6 is homologous to PD-L1 or B7-H3 [7,9]. B7-H6 is primarily expressed in humans and rats as a surface protein. The surface form of B7-H6 binds to NKp30 expressed on NK cells and induces NK cell activation. In the soluble form of B7-H6, the metalloproteases disintegrin and metalloproteases (ADAM)-10 and ADAM-17 shed the ectodomain of B7-H6, which inhibits NK cell function by inhibiting NKp30 activation. In NK cells chronically stimulated with the soluble form of B7-H6, the cognate receptor of B7-H6, NKp30, was downregulated [10].

This study investigated the clinical advances associated with B7-H6. We explored the expression of B7-H6 across cancers, pathophysiological conditions that induce its expression, intrinsic and extrinsic pathways regulating its activation, and emerging targeted anticancer therapies. Understanding the mechanism of action of B7-H6 will provide valuable insights into its potential as a therapeutic target, advancing the development of both B7-H6-specific and broader B7 family-based therapies.

## 2. Expression of B7-H6

B7-H6 is expressed in various cancer types. When it was first identified, B7-H6 was expressed in the tumor cells of patients with lymphoma and various cell lines, including T lymphoma, B lymphoma, myeloid leukemia, and melanoma, but it was rarely expressed in normal peripheral blood mononuclear cells [7,9]. It was previously thought that B7-H6 is a cancer-specific ligand, as its expression was rarely detected in normal cells compared to cancer cells. However, it has since been shown that B7-H6 is expressed not only in cancer cells but also in normal cells under special circumstances, such as in infected cells or activated T cells. Below, we describe the different pathophysiological contexts in which B7-H6 is expressed.

### 2.1. Cancer

Since its identification in clinical lymphoma samples, liquid cancer cell lines, including B lymphoma, T lymphoma, and myeloid leukemia, and solid cancer cell lines, including melanoma, colon carcinoma, and cervical carcinoma [7], several studies have confirmed the expression of B7-H6 in various types of liquid and solid cancer samples and cell lines, including cervical cancer, hepatocellular carcinoma, glioma, non-Hodgkin lymphoma, T-cell lymphoblastic lymphoma, and diffuse large B-cell lymphoma. Furthermore, the expression of B7-H6 has shown strong associations with patient prognosis and cancer progression. The following sections cover the outcomes of studies related to each cancer type.

#### 2.1.1. Liquid Cancer

##### Acute Myeloid Leukemia (AML)

Baragano Raneros et al. identified elevated B7-H6 expression in patients with AML [11]. Elevated levels of B7-H6 were induced by bromodomain-containing protein 4 (BRD4), which is overexpressed in AML. Patients with high B7-H6 expression showed poor prognosis, suggesting that B7-H6 is a therapeutic target.

##### Chronic Myeloid Leukemia (CML)

BCR-AML1 is a major oncogene in patients with CML, in which it induces uncontrolled cell proliferation. The level of BCR-AML1 has been associated with CML progression [12]. Cao et al. found that patients with CML expressing BCR-ABL1 also express B7-H6 [13]. Moreover, patients expressing B7-H6 showed longer progression-free survival.

##### T-Lymphoblastic Lymphoma

B7-H6 was expressed in 61.5% of patients with T-lymphoblastic lymphoma [14], and membrane and cytoplasmic expression was detected in 38.5% of patients. B7-H6 expression did not affect survival rate but was associated with prognostic factors in T-lymphoblastic lymphoma (B symptom, high ECOG score, elevated serum lactate dehydrogenase level) and reduced complete remission. In particular, because mRNA and protein levels are induced after chemotherapy, the expression of B7-H6 seems to correlate with the chemotherapy response [15].

##### Non-Hodgkin Lymphoma (NHL)

Wu et al. demonstrated that B7-H6 was presented in B-cell NHL, and the downregulation of B7-H6 inhibited cancer progression and reinforced chemosensitivity against vincristine and dexamethasone [16]. The induction of B7-H6 in B-NHL cells increased proliferation, colony formation, migration, and invasion. Yang et al. identified B7-H6 expression in B-cell NHL, T-cell NHL patient samples, and cell lines [17]. In B- and T-NHL cells, the presentation of B7-H6 promoted proliferation, migration, and invasion through the Ras/MEK/ERK pathway.

#### 2.1.2. Solid Cancer

##### Oral Squamous Cell Carcinoma

B7-H6 was detected in patients with oral squamous cell carcinoma but not in normal oral mucosa [18]. The B7-H6 level was related to the degree of cancer differentiation but not with age, sex, tumor size, lymph node metastasis, clinical stage, or recurrence. Although the B7-H6 level was irrelevant to overall survival (OS) and disease-free survival rates, it showed an association with the 5-year survival rate.

##### Hepatocellular Carcinoma

Immunohistochemistry (IHC) revealed B7-H6 in samples of hepatocellular carcinoma patients [19]. Higher B7-H6 expression has been associated with reduced tumor size and survival rates. Similarly, B7-H6 was negatively correlated with OS rates [19,20,21]. The downregulation of B7-H6 inhibited cancer cell proliferation, induced apoptosis, and arrested the cell cycle in the G1 phase. However, the overexpression of B7-H6 did not have any effects on the clinicopathological parameters.

##### Small-Cell Lung Cancer (SCLC)

A total of 56% of the 103 SCLC patient samples exhibited B7-H6, as confirmed by IHC, whereas normal lung tissues did not [22]. Zhang et al. showed that a higher level of B7-H6 was found in the early and non-distant metastasis stages of SCLC [22]. Moreover, patients with high B7-H6 levels showed dramatically worse survival rates in the first 2 years, indicating that B7-H6 activation may be related to the early disease stage. However, Thomas et al.’s study showed the opposite results to Zhang et al.’s study. Thomas et al. showed that B7-H6 was expressed at a higher level than PDL-1, and high levels of B7-H6 resulted in longer progression-free survival and increased total immune infiltrate levels in 39 patients [23]. In addition, gene expression of B7-H6 was related to a weaker gene signature for NK cell activation. Thomas et al. interpreted these results as the interaction of B7-H6 expression with an immune signature. Of the 103 patients in Zhang et al.’s study, 76 (74% of all patients) had limited-stage SCLC, whereas of the 39 patients in Thomas et al.’s study, only 14 (36% of all patients) had a limited stage, and the remaining 64% of patients had an extensive stage. Thus, the effect of B7-H6 on survival may differ depending on the stage of the SCLC patient. In conclusion, B7-H6 is a potential predictive marker and therapeutic target in SCLC.

##### Non-Small-Cell Lung Cancer (NSCLC)

The expression of B7-H6 was not significantly different between cancerous and non-cancerous tissues in NSCLC [24]. There was no correlation between B7-H6 expression and survival rate or age; sex; tumor size; histological classification; lymphocyte node metastasis; tumor, node, and metastasis (TNM) stage; or distant metastasis. Therefore, B7-H6 appears to have limited clinical relevance in NSCLC.

##### Cervical Squamous Cell Carcinoma

IHC showed a progressive increase in the expression of B7-H6 with the development of precancerous lesions into cervical cancer, suggesting that its expression is associated with cancer development [25]. The presentation of B7-H6 was related to stromal invasion, lymph vascular space involvement, lymph node metastasis, and International Federation of Gynecology and Obstetrics (FIGO) stage [26]. Moreover, the peripheral blood concentration of the soluble form of B7-H6 was higher in patients with cervical squamous cell carcinoma than in healthy controls.

##### Ovarian Cancer

In ovarian cancer, IHC confirmed a strong relation between B7-H6 expression and distant metastasis status and FIGO stage [27], which was not the case for age, tumor size, tumor location, pathological stage, or nodal metastasis. Patients with lower levels of B7-H6, compared to higher levels, had significantly superior survival rates. In conclusion, the presentation of B7-H6 is considered to be positively related to ovarian cancer progression and metastasis.

##### Pancreatic Cancer (PC)

The surface expression of B7-H6 has been found in PC tissues but not in normal tissues [28]. Also, a soluble form of B7-H6 was discovered in the sera of patients with PC, in which its expression showed a relationship with the TNM stage. The levels of both B7-H6 types were related to poor prognosis in patients with PC. Moreover, higher levels of B7-H6 in malignant tissues and sera correlated to shorter survival rates. Analysis of a dataset from The Cancer Genome Atlas (TCGA) confirmed the increased expression of B7-H6 in PC tissues [29].

##### Esophageal Squamous Cell Carcinoma

A total of 91.72% of patients with esophageal squamous cell carcinoma revealed a presentation of B7-H6. The level of B7-H6 expression was associated with T stage, lymphatic metastasis status, and worse OS [30].

##### Breast Cancer

In breast cancer, the level of B7-H6 was associated with the increased expression of human epidermal growth factor receptor 2 [31]. Higher B7-H6 levels were also associated with shorter survival and lymph node metastasis, suggesting that its expression is associated with cancer progression and invasion.

##### Gastric Carcinoma/Colorectal Cancer

In a TCGA dataset analysis, both gastric carcinoma and colorectal cancer showed elevated levels of B7-H6 [29]. The surface expression of B7-H6 in gastric cancer cells was confirmed by IHC.

##### High-Risk Neuroblastoma

Serum concentrations of soluble forms of B7-H6 in high-risk neuroblastoma were associated with the downregulation of NKp30 expression, bone marrow metastasis, and chemoresistance [32]. The increasing expression of B7-H6 was associated with increasing NK cell activation via NKp30 expression, corresponding to higher soluble forms of B7-H6 levels in the sera of patients with high-risk neuroblastoma.

##### Astrocytoma

The surface expression of B7-H6 in astrocytoma patients was confirmed using IHC [33]. B7-H6 exhibition was associated with the World Health Organization grade but not with survival rates.

##### Glioma

In glioma cell lines, the level of B7-H6 presentation was linked to disease development [34]. The B7-H6 protein was expressed at high levels in glioma tissue but not in normal cells, and B7-H6 expression showed a significant relevance to cancer progression and pathological type [35]. The downregulation of B7-H6 expression increased the viability, migration, and proliferation of glioma cell lines. B7-H6 showed preferential expression in glioma stem-like cells, which enhanced cancer cell proliferation [36].

### 2.2. CD14+CD16+ Pro-Inflammatory Monocytes and Neutrophils

The B7 family, along with B7-H6, is induced by pro-inflammatory stimuli. Matta et al. showed that B7-H6, similar to the rest of the B7 family, is induced under pro-inflammatory conditions [37]. Specifically, B7-H6 was increased in circulating pro-inflammatory CD141CD161 monocytes from patients with sepsis [37]. We found that the B7-H6 transcript was induced upon stimulation of peripheral blood mononuclear cells with Toll-like receptor (TLR) agonists [37]. Finally, we showed that B7-H6 expression was induced in CD141CD161 pro-inflammatory monocytes and neutrophils by stimulation with the ligands of Toll-like receptors or pro-inflammatory cytokines, such as interleukin (IL)-1b and tumor necrosis factor-alpha (TNF-α) [37].

### 2.3. Activated T Cell

Kilian et al. showed that activated T cells transiently express B7-H6 to induce NK cell-mediated lysis [38]. In addition to the activating ligand B7-H6, the levels of the inhibitory ligands, HLA-E and CLEC2D, were also reduced. This mechanism allows NK cells to target themselves, unlike other immune checkpoint inhibitors that, upon activation, prevent excessive or prolonged T-cell activation through downstream signaling within T cells. The expression of B7H6 in pro-inflammatory monocytes, neutrophils, and activated T cells showed that B7H6 is expressed not only in cancer cells but also in normal cells. It appears to be a part of the innate immune system that is recognized and killed by NK cells, the innate immune cells, when cells are in a state of inflammation or hyperactivity. However, B7H6 is still rarely expressed in normal cells and is mostly expressed in cancer cells, making it a prime target for cancers.

## 3. Upstream of B7-H6

**Figure 1:**
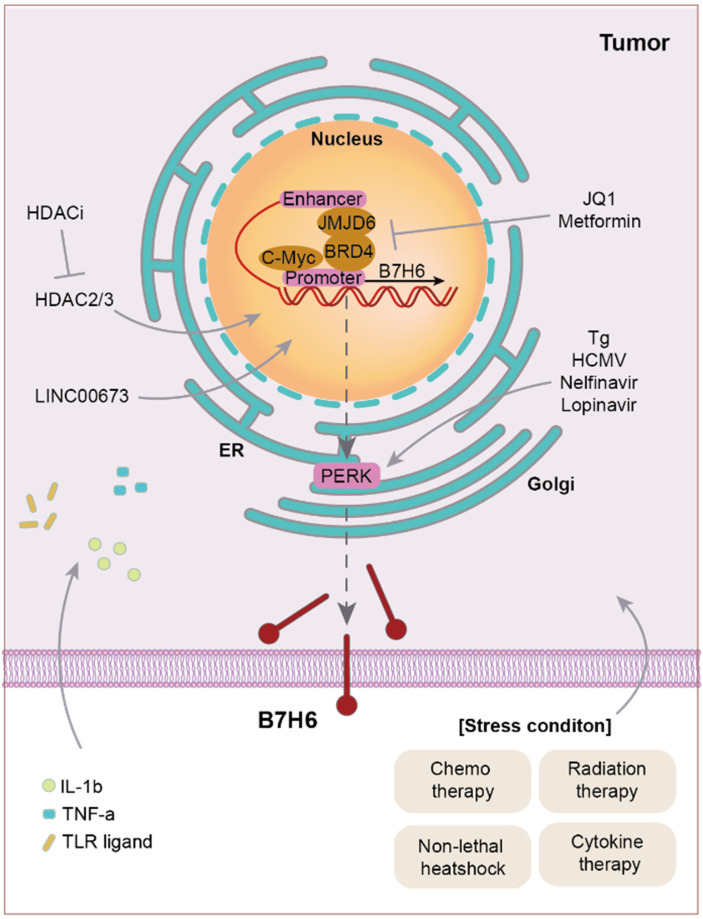
Different pathophysiological conditions that induce B7-H6 expression. B7-H6 is upregulated under stress conditions (chemotherapy, radiotherapy, non-lethal heat shock, and cytokine therapy), TLR ligand treatment, PERK induction (Tg, HCMV infection, nelfinavir, and lopinavir), and HDAC2/3 upregulation. c-Myc and BRD4 bind to the promoter site of B7-H6. BRD4 co-binds with JMJD6 to promote *B7-H6* mRNA transcription.

B7-H6 is rarely expressed in normal cells outside specific situations such as cancer, inflammation, and T cell activation. Cao et al. reported that various cancer therapeutics, including chemotherapy (cisplatin, 5-fluorouracil), radiation therapy, non-lethal heat shock, and cytokine therapy (TNF-a), act as stress inducers and upregulate the expression of B7-H6 (Figure 1) [15]. Moreover, the integrated stress response (ISR)—the phosphorylation and downstream activation of the translation initiation factor elF2—induces B7-H6 expression [39]. elF2 is phosphorylated under various stress conditions, and elF2 phosphorylation and ISR are induced by some drugs. The clinically approved HIV protease inhibitors nelfinavir and lopinavir, ER stress inducer thapsigargin (Tg), and human cytomegalovirus (HCMV) infection upregulate B7-H6 through ISR. Specifically, the ISR-mediated induction of B7-H6 requires protein kinase R-like ER kinase (PERK), a canonical sensor of the unfolded protein response.

Many classes of molecules can induce B7-H6 expression. TLR ligands and inflammatory cytokines, including IL-1b and TNF-α, induced B7-H6 expression in CD141CD161 inflammatory monocytes and neutrophils [37].

The upregulation of class 1 histone deacetylase (HDAC)3 concomitantly increased the expression of B7-H6, which was suppressed by an HDAC inhibitor (HDACi) [40]. Furthermore, the downregulation of B7-H6 expression via HDACi treatment reduced NKp30-dependent effector function. c-Myc expression upregulated B7-H6 levels in various carcinomas, including melanoma, PC, and neuroblastoma cell lines, among others [41]. In the primary tissues of hepatocellular carcinoma, lymphoma, and NB, the mRNA level of c-Myc was positively correlated with B7-H6 expression. Since HDAC3 can trigger cancer proliferation, metastasis, and angiogenesis [42] and c-Myc is a well-known pro-tumorigenic molecule, the relationship between HDAC3, c-Myc, and B7-H6 is of particular clinical interest. LINC00673, a long non-coding RNA, was significantly upregulated in BC and induced the expression of B7-H6, leading to cancer cell proliferation, metastasis, and epithelial–mesenchymal transition (EMT) [43].

The promoter region of B7-H6 has been described. One of the BET family members, bromodomain-containing protein 4 (BRD4), binds to the hyperacetylated regions of the *B7-H6* promoter to recruit transcriptional machinery and regulate the expression of B7-H6 [11]. BRD4 then co-binds to the distal enhancers of JMJD6 and B7-H6, suggesting that *B7-H6* is transcriptionally paused and activated during malignant transformation. JQ1, a BET protein inhibitor, and metformin, a metabolic inhibitor, interfere with the assembly of transcriptional machinery. As BRD4 is highly expressed in patients with AML, this suggests a correlation between B7-H6 and BRD4 expression in patients with AML and potential roles in tumorigenesis.

## 4. Intrinsic Pathways of B7-H6

B7-H6 activates pathways intrinsic to B7-H6-expressing cancers, affecting various functions. B7-H6 induces cancer cell proliferation, migration, invasion, and anti-apoptosis in various cancers, including glioma, lymphoma, and T-lymphoblastic lymphoma [14,17,34]. The downregulation of B7-H6 in lymphoma cell lines reduced cancer cell proliferation and colony formation and drastically reduced the stemness and migration properties of glioblastoma cancer cells [44]. In vivo, cancers with downregulated B7-H6 expression also showed reduced proliferation and metastasis [21].

**Figure 2:**
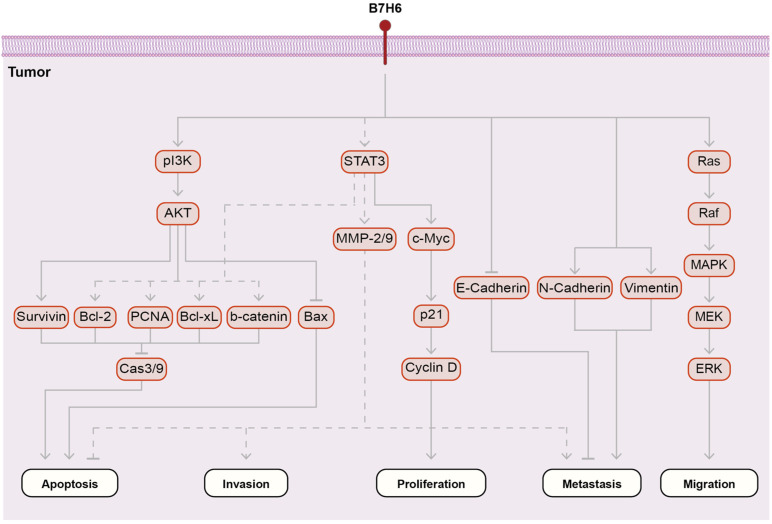
Intrinsic pathway of B7-H6. B7-H6 activates the PI3K/AKT, STAT, and Ras/MEK/ERK pathways through phosphorylation. The downstream signaling of B7-H6 promotes the anti-apoptotic, invasion, proliferation, metastasis, and migration abilities of cancer cells.

B7-H6 induced the phosphorylation of MEK, ERK, and HIF-1 in the Ras/MEK/ERK pathway (Figure 2) [17,45]. This promotes the translocation of activated ERK1/2 to the nucleus to improve cancer cell survival, proliferation, migration, and differentiation [17,45]. The downregulation of B7-H6 in glioma stem cells inactivated the phosphatidylinositol-3-OH kinase (PI3K)/AKT and ERK/MAPK signaling pathways [36,45].

Treatment with a p38 MAP kinase inhibitor, JNK inhibitor II, PI3K inhibitor, and MEK inhibitor inhibited the expression of B7-H6. The downregulation of B7-H6 altered the phosphorylation pattern of HIF-1 and activation of Ras signaling in the NHL cell lines Jurkat and Raji and inhibited the phosphorylation of MEK1/2 and ERK1/2 via the KEGG pathway, as evidenced by an enrichment analysis of altered phosphoproteins [17].

B7-H6 activates the PI3K/AKT pathway. The downregulation of B7-H6 expression in glioma stem-like cells deactivated the PI3K/AKT signaling pathway [36,45] and promoted cell proliferation, migration, invasion, and metastasis in glioma by decreasing matrix metalloproteinase (MMP)-2, MMP-9, Vimentin, N-cadherin, and Survivin levels and increasing E-cadherin and Bax levels [34]. In EMT, E-cadherin is replaced with N-cadherin. The decrease in B7-H6 levels led to a decrease in N-cadherin and an increase in E-cadherin levels, implying that B7-H6 is involved in the EMT process.

MMP-2 and MMP-9 levels were reduced by the downregulation of B7-H6 expression in glioma and B-cell NHL [16]. MMPs are calcium-dependent zinc endopeptidases involved in extracellular matrix degradation and remodeling. MMP-2 and MMP-9 are also involved in cell death by destroying basilar membrane molecules and have been implicated in cancer metastasis and invasion [46,47]. Thus, the decreased expression of MMP-2 and -9 due to the downregulation of B7-H6 inhibits cancer cell death, metastasis, and invasion.

Survivin expression was also reduced following the downregulation of B7-H6 expression in glioma and B-cell NHL [16,34]. Survivin is an inhibitor of the apoptotic protein family and is highly expressed in cancers; therefore, a decrease in Survivin levels indicates the induction of apoptosis [48,49]. Survivin is an important molecular marker of the cell cycle [16]. The downregulation of B7-H6 expression reduced the levels of PCNA, a marker for the S phase, and Survivin, a marker for the G2/M phase [26,27]. Thus, B7-H6 is involved in the cell cycle via the regulation of PCNA and Survivin.

B-cell lymphoma (Bcl)-2/Bcl-xL prevents apoptosis by inhibiting the release of pro-apoptotic molecules into the cytosol [29,30]. Bax is an important BCL-2 family member that promotes apoptosis, in contrast to Bcl-2/Bcl-x1 [50]. The decreased expression of Bcl-2 and increased expression of Bax indicated that the downregulation of B7-H6 expression triggers apoptosis [34].

The STAT3 pathway induces various responses in cancers, including proliferation, cell cycle progression, and metastasis [51]. B7-H6 induces the activation of STAT3, resulting in various tumorigenic responses. The downregulation of B7-H6 expression in glioma stem-like cells resulted in decreased RNA guanine-7 methyltransferase (RNMT) and decreased Myc levels [36]. The downregulation of B7-H6 expression reduced c-Myc expression in a lymphoma cell line [16].

B7-H6 downregulation decreased the levels of cyclin D1, CDK4, CDK6, and pRb proteins, increased the expression of Rb, and increased the expression of the CDK inhibitor P21. The downregulation of B7-H6 protein levels decreased c-Myc, c-Fos, and cyclin-D1 expression in glioma cells [34]. Cyclin D1 forms a complex with CDK4/6 and phosphorylates Rb, inactivating it. Phosphorylated Rb promotes the G1/S transition [34]. Finally, the knockdown of B7-H6 in a triple-negative breast cancer cell line increased β-catenin, caspase-3, and Bax levels and decreased SMAD4 and Bcl-2 levels [52].

The SH2 and SH3 domains in the endodomain of B7-H6 are activated by protein kinases [17,53]. However, how the endodomain of B7-H6 triggers the intrinsic pathway remains unclear.

Collectively, these findings indicated that B7-H6 induces tumorigenesis through multiple pathways, including the Ras/MEK/ERK, PI3K/AKT, and STAT3 pathways.

## 5. Extrinsic Pathway of B7-H6

### 5.1. NKp30

The NCR family represents type-1 transmembrane proteins expressed on the plasma membrane of immune cells [54]. NKp30 was identified in 1999 by Pende et al. as a member of the NCR family (including NKp30, NKp44, NKp46, and NKp80) of activating receptors expressed on NK cells [55,56]. NKp30 is expressed on all mature resting and activated NK cells and promotes NK cell activities. In addition to NK cells, NKp30 is expressed on Vdelta1^+^, CD8^+^, and UCB T cells stimulated with IL-15 [57,58,59]. Owing to alternative splicing, there are three isoforms of NKp30: NKp30a, NKp30b, and NKp30c. NKp30a and NKp30b trigger IFN-r production and induce its activation. NKp30c, unlike other isoforms, produces very little IFN-r but induces the secretion of the immunosuppressive cytokine IL-10 [54,60]. This is believed to be because NKp30c interacts poorly with CD3z [56].

### 5.2. Ligands of NKp30

The activating ligands for NKp30 include B7-H6, heparan sulfate GAGs (HS-GAGs), cytomegalovirus, *Plasmodium falciparum* erythrocyte membrane protein (PfEMP1), and BAT3/BAG6, among others [7,54,61,62,63,64,65,66]. However, B7-H6 is the only molecule expressed on the cell membrane and induces the formation of an immune synapse between NK cells and target cells, promoting the killing effect. HS-GAGs bind to all NCR family members, including NKp30. NKp30 ligands not only act independently but can interact with multiple ligands. In CLL, the interaction of NKp30 with BAG6 has been studied for its anticancer effect [60,67].

The inhibitory ligands of NKp30 include haemagglutinin, pp65, galectin-3, and PfEMP1 [68,69,70]. galectin-3, which is expressed in cancer cells, is a soluble ligand that binds to NKp30 and inhibits NKp30-mediated activation and cytolysis [67]. galectin-3 expression inhibits cancer proliferation in neuroblastoma and is associated with better prognosis [70]. Notably, there is no obvious structural similarity between the ligands for NKp30. It remains unclear what ligands NKp30 recognizes.

### 5.3. Structure of NKp30

NKp30 (molecular weight of 30 kD) consists of an ectodomain containing an Ig-like fold, stalk domain, transmembrane domain, and endodomain-containing structures related to signaling pathways, such as immunoreceptor tyrosine-based activation motif (ITAM), CD3Z, and FcR [54,55,56,60]. The stalk domain appears to play an important role in receptor activity by increasing ligand-binding affinity [56,71]. This 15-residue domain is highly sensitive to point mutations. One residue in this domain is the cationic arginine Arg143, which is normally located on the extracellular membrane surface; however, upon ligand binding, it is translocated into the membrane to enhance the interactions of CD3Z or FcR [56,64,72].

### 5.4. NK Lysis through NKp30

**Figure 3:**
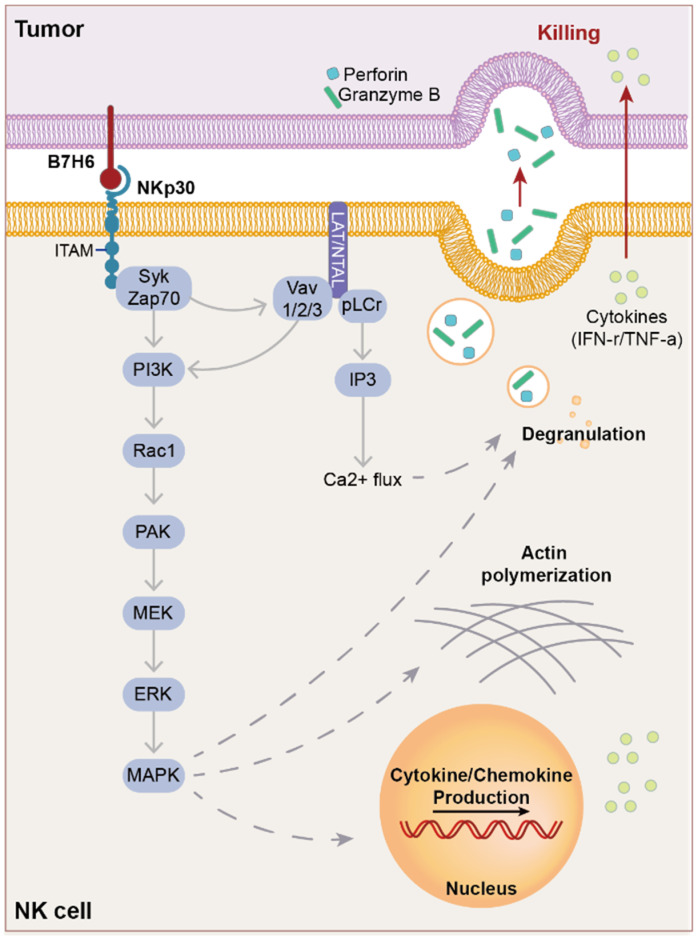
Extrinsic pathway of B7-H6. When B7-H6 binds to its cognate receptor NKp30, NKp30 activates NK cells and induces target lysis. ITAM is the end domain of NKp30 phosphorylates Syk and Zap70, in turn activating a cascade from the PI3K to MAPK pathway. LAT/NTAL contributes to the activation of the NKp30 pathway. Finally, NKp30 activation induces cytokine/chemokine production, actin polymerization to form the immune synapse, and cytotoxic granule (perforin and granzyme) production, resulting in target lysis.

Activating receptors on NK cells, including NKp30, induce NK cell activation via ITAM [56]. Upon binding of the cognate ligand to the activating receptor, ITAM is phosphorylated by the Src family members (Figure 3), thereby promoting the recruitment and activation of Syk and Zap70. The activated tyrosine kinases activate several molecules, including phospholipase C (pLCr), PI3K, and Vav1/2/3, among others [56,73]. pLCr induces Ca^2+^ influx and recruits PI3K and Vav1, resulting in the recruitment of small G protein Rac 1, which induces cascade phosphorylation through the PAK1-MEK-ERK signaling pathway, subsequently activating the MAPK signaling pathway [74,75,76,77,78]. This cascade ultimately leads to a cytotoxic NK cell response that includes actin skeleton rearrangement, cytotoxic granule release, and cytokine/chemokine production [73]. Upon activation by B7-H6, NKp30 secretes cytotoxic mediators (TNF-α, IFN-r, Perforin, and granzymes) [56]. The common signaling pathways of the NCR family have been partially described; however, the mechanisms underlying NKp30-specific NK cell activation remain unclear [73].

## 6. Therapy Targeting B7-H6

**Figure 4:**
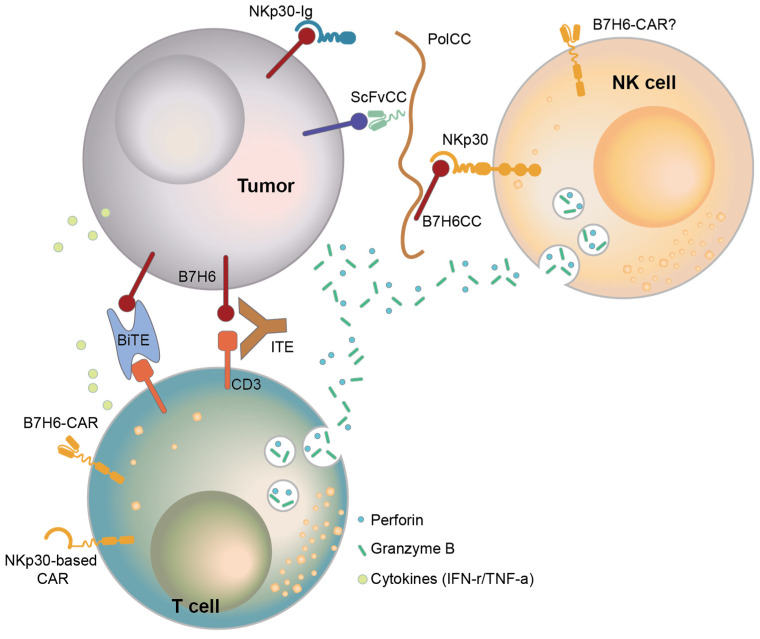
Cancer therapies targeting B7-H6. B7-H6-scFv or NKp30-based CAR-T cells target cancers expressing B7-H6. BiTE and ITE therapies co-bind the cancer-expressed T-cell marker CD3 to B7-H6, inducing the formation of a robust immune synapse between the cancer and T cells. Treatment with the NKp30-Ig protein, a cognate receptor of B7-H6, inhibits cancer growth and induces macrophage ADCC. A protein–polymer complex made by binding B7-H6 and a cancer-specific scFv protein to a polymer can activate NK cells while recruiting NK and cancer cells into the same space. To avoid T cell fratricide of CAR-T cells and avoid targeting normal NK cells, CAR-NK cells targeting B7-H6 are an alternative to B7-H6-CAR-T cell therapy.

Since the identification of B7-H6 as a cancer antigen, several targeted immunotherapies have been developed. One method involves treatment with a monoclonal antibody or Ig fusion protein that specifically binds to the cancer antigen (Figure 4). Treatment of a prostate cancer cell line with NKp30-Ig reduced cancer growth, and macrophages activated against NKp30-Ig-coated cells mediated antibody-dependent cell cytotoxicity (ADCC) [79].

Bispecific T-cell engagers (BiTEs) are engineered to simultaneously bind an antibody targeting one cancer antigen and one CD3 molecule [80]. Unlike natural antibodies, BiTEs recruit T cells to engage target cells. Wu et al. developed a B7-H6-specific BiTE to enhance cancer elimination and host anticancer immunity and found that it increased the survival of RMA/B7-H6 lymphoma-bearing mice through perforin and IFN-r effector mechanisms [81].

Although BiTEs show high anticancer potency, their short half-life must be compensated by inconvenient dosing regimens. Consequently, IgG-like T-cell engager (ITE) therapy has been developed to improve dosing convenience. ITEs are designed to simultaneously bind a target expressed on cancer cells and a marker expressed on T cells by connecting two monovalent antigens that bind to each antigen with a flexible peptide linker. Zhang et al. developed an ITE monotherapy that targets B7-H6/CD3 [29] that facilitated the formation of cytolytic synapses between T and cancer cells, thereby increasing the cytotoxicity and infiltration of T cells, resulting in cancer regression. Zhang et al. proposed a combination therapy of ITE and anti-PD-1 since anti-PD-1 also increased IFN-r and IL-2 levels and enhanced cytotoxicity. The clinical applicability of this ITE is currently being investigated in a non-randomized open-label dose-escalation trial (NCT04752215) as monotherapy and in combination with the anti-PD-1 antibody ezabenlimab.

Because B7-H6 is widely expressed in various types of cancers and rarely expressed in normal tissues, CAR-T cells targeting B7-H6 have been studied. Zhang et al. developed NKp30-based CAR-T cells and showed that they increased T-cell effector function in vivo [82]. In particular, the expression of chimeric NKp30, including the CD28 stalk and CD3Z domains, showed the best outcome and was associated with the highest survival rates in vivo [83].

Instead of NKp30-based CAR-T cells, B7-H6 CAR-T cells using single-chain Fv (scFv) have been developed to target B7-H6. From a human scFv library, an scFv with good affinity for directly targeting B7-H6 was selected, and B7-H6-directed CAR-T cells expressing the selected scFv were constructed and evaluated for efficacy [39,81,83,84,85]. In several studies, CAR-T targeting of B7-H6 has shown excellent anticancer activity in vitro and in vivo against various cancers.

T cells armed with the bispecific antibody anti-CD3×anti-B7-H6 (B7-H6 Bi Ab) showed high cytotoxicity against blood cancer cells, increased the Granzyme B and Perforin levels, and produced high levels of T cell-derived cytokines (TNF-α, IFN-r, IL-2) while also increasing the levels of the activation marker CD69 [53].

The development of monoclonal antibodies, recombinant proteins, and cell-based therapies has greatly advanced; however, these treatment modalities are limited by their low efficiency in reaching the target. Kalouskova et al. developed a protein–polymer complex that can simultaneously achieve NK cell activation and cancer targeting [86]. The coil was attached to a B7-H6 protein that activates NK cells and an antibody against a cancer-targeting marker to form a complex with the polymer. This complex enabled the activation of NK cells and the recruitment of both cancer and NK cells within the same environment. Consequently, the protein–polymer complex using the B7-H6 protein is expected to show improved therapeutic efficacy and in vivo stability against cancer.

As described, therapies targeting B7-H6 have evolved in several directions due to the nature of B7-H6 as a cancer-specific target. However, its low expression levels in cancer cells pose a challenge for developing effective treatments. To overcome this, strategies should focus either on increasing B7-H6 expression in tumors or on enhancing the sensitivity of therapies designed to detect and eliminate B7-H6-expressing cells.

One approach is to combine conventional treatments, such as chemotherapy or radiation, with B7-H6-targeted therapies. Chemotherapeutic agents, like cisplatin and 5-fluorouracil, or radiation therapy, not only kill cancer cells but can also induce B7-H6 expression in any surviving cancer cells [15]. By first applying these conventional therapies to reduce the tumor burden and then using B7-H6-targeted treatments for the remaining cancer cells, it may be possible to achieve greater efficacy with fewer side effects.

In cell-based therapies, CAR-NK cells offer a promising alternative to CAR-T cells for targeting B7-H6 (Figure 4). Since activated CAR-T cells express high levels of B7-H6 [38], they may trigger attacks from the patient’s NK cells or even cause fratricide among themselves. In contrast, CAR-NK cells can avoid these issues. B7-H6-targeting CAR-NK cells can detect B7-H6 through both CAR and NKp30, a receptor naturally expressed by NK cells, which may enable more sensitive recognition and destruction of B7-H6-expressing cancer cells.

## 7. Conclusions

The discovery of B7-H6 as a member of the B7 family has opened new avenues for cancer-specific therapies. Unlike other B7 family members, B7-H6 is rarely expressed in normal tissues but is upregulated in a variety of cancers, making it an ideal target for precision therapies. Its interaction with NKp30 on NK cells plays a key role in immune surveillance and cancer immune evasion. By understanding the regulatory mechanisms behind B7-H6 expression, as well as its intrinsic and extrinsic pathways, researchers can develop therapies that not only target B7-H6 directly but also enhance the broader immune response against cancers. Current efforts to target B7-H6, either through monoclonal antibodies or engineered immune cells, show promising potential for more effective and less toxic cancer treatments. With continued progress, B7-H6-based therapies may contribute greatly to the landscape of molecular targeted therapy and immunotherapy, especially for improving treatment outcomes while minimizing side effects. Further exploration of the roles of B7-H6 in cancer biology may lead to innovative strategies that exploit its cancer-specific properties and advance treatment precision and patient outcomes.
